# Association between disability and periodontal diseases in older adults

**DOI:** 10.6026/973206300220322

**Published:** 2026-01-31

**Authors:** Anushree Tiwari, Archana Patel, Shubhra Raghav, Rahul Katiyar, Aparna Dave, Hemal Patel, Santosh Kumar

**Affiliations:** 1 Independent Researcher, Paoli, Pennsylvania, 19301, USA; 2 Department of Pharmacology, Smt. B. K. Shah Medical Institute & Research Center, Sumandeep Vidhyapeeth, Waghodia, Vadodara, Gujarat, India; 3 Department of Public health Dentistry, Kothiwal Dental College & Research Centre, Moradabad, Uttar Pradesh, India; 4 Department of Periodontology, Maharana Pratap Dental College, Kothi, Mandhana, Kanpur, Uttar Pradesh, India; 5 Department of Oral Pathology, Faculty of Dental Sciences, SGT University, Gurugram, Haryana, India; 6 Department of Public Health Dentistry, Faculty of Dental Science, Dharmsinh Desai University, Nadiad, Gujarat, India; 7 Department of Periodontology & Implantology, Karnavati University, Uvarsad, Gandhinagar, Ahmedabad, Gujarat, India

**Keywords:** Functional disability, periodontal disease, older adults, plaque index, probing depth, clinical attachment loss

## Abstract

Periodontal diseases and functional disability are important issues of collective health among the aging groups of people. Therefore, it is of interest to establish the relationship between disability status and prevalence and severity of periodontal diseases in community- based older adults aged 65 years and above. Hence, a total of 384 of them received a complete periodontal examination and disability evaluation based on Barthel Index and Lawton Instrumental Activities of Daily Living scale, with the disability being none, mild, moderate and severe. Findings showed a much higher prevalence of moderate-to-severe periodontitis in the disabled (78.3 percent) than in non-disabled persons (52.1 percent, p=0.001) and also that multivariate analysis showed that severe disability independently predicted periodontal disease (OR=3.84, 95 percent CI: 2.17-6.79, p=0.001) after controlling confounders. Thus, we show the urgent necessity of the combination of geriatric care that would help to prevent the cyclical decline of aging groups by targeting both functional capacity and oral health preservation.

## Background:

The world is facing unprecedented burden in terms of population aging and this has posed major challenges to the healthcare systems around the world with the projections showing that the number of people aged 65 years and above will be 16% of the global population by 2050 [1]. Such a demographic change requires holistic knowledge of the interconnected health issues with a disproportionate presence in older adults such as functional disability and periodontal diseases [2]. Functional disability, which refers to impairment or reliance of simple and instrumental activities of daily living, impacts about 40 percent of elderly living in the community and is a major quality of life, health care service and death determinant in older adults [3]. Periodontal diseases include gingivitis and periodontitis, which are chronic inflammatory diseases that involve the supporting structures of teeth and its prevalence is more than 70 percent of adults at age 65 years and above [4]. Severe periodontitis is the sixth widespread disease in the world with an estimated incidence of 743 million individuals, with serious effects to loss of teeth, masticatory impairment, nutritional disability and complications to the systemic health [5]. Periodontal disease is an inflammatory process that is mediated by dysbiotic microbial communities and host immune response regulated by the immune system, which has been associated with a wide range of systemic disorders such as cardiovascular disease, diabetes mellitus and cognitive impairment [6]. The possibility of the bi-directional relationship between disability and periodontal disease is a strong field to research. Oral health capacity, dental health care access and nutritional adequacy may be impaired by functional restrictions, which expose them to the risk of periodontal disease [7]. On the other hand, tooth loss and masticatory deficiency caused by periodontal disease can lead to malnutrition and sarcopenia and consequently, functional degradation, which form a vicious cycle of deterioration [8]. This association is essential to understand in order to come up with combined preventive measures and care models of aging populations.

Some of the areas that have been explored through the use of recent epidemiological research include aspects of oral health among disabled populations. It has been proven that physical constraints are related to poor oral health outcomes, such as dental caries, edentulism and self-reported oral health problems [9, 10]. The prevalence rates and severity scores of periodontal disease among older adults with functional impairments have been found to be high in studies that specifically examined this disease in that group [11]. A lot of studies, however, have had small sample sizes, have not been served with a full-fledged periodontal analysis or have not been able to correct against significant confounding variables like smoking, diabetes and socioeconomic factors [12]. Also, past literature has mostly examined institutionalized elderly subjects or those with certain disabilities and cannot be generalized to older adults living in community with different levels of functional impairments [13]. There is further heterogeneity in disability measurement tools and periodontal disease measures in studies, which makes it even more difficult to synthesize existing evidence [14]. Most significantly, there have been very minimal researches that have utilized multidimensional disability measurement that entails both basic and instrumental activities of daily living and at the same time, perform rigorous clinical periodontal examination in line with established standards. Therefore, it is of interest to examine how functional disability is linked to prevalence and severity of periodontal diseases among community-dwelling adults aged 65 years and above, as well as to determine independent predictors of periodontal disease among adults living in the community and aged 65 years and above.

## Materials and Methods:

## Study design and setting:

The study was a cross-sectional observational study that was done between March 2024 and July 2024 in three community health centers in urban areas. The Institutional Review Board approved the study protocol (Protocol No. 2024-GER-073) and all participants signed a written informed consent before taking part in the study. The research was conducted using guidelines of Strengthening the Reporting of Observational Studies in Epidemiology (STROBE).

## Sample size and participant selection:

Calculation of sample size was done by using openEpi software version 3.01 whereby; prevalence of moderate periodontitis in non- disabled older adults was assumed to be 55 percent, odds ratio of associated disability-periodontitis was expected to be 2.0, 80 percent statistical power was assumed, 95 percent confidence level was assumed and ratio of disabled to non-disabled participants was 1:1. This computation revealed that there was a minimum number needed (348 participants), which was raised to 400 to counter this possibility of incomplete data and the final analysis figure was 384 fully assessed participants.

The adults aged 65 years and older living in the community were recruited by sampling the convenient community health centers, senior citizen associations and residential complexes with snowball sampling up to the age of 75 years. Inclusion criteria indicated: (1) 65 years of age or older, (2) no less than 8 natural teeth, (3) living in the community, (4) having informed consent or having legal guardian consent, (5) readiness to receive clinical examination. Those individuals who were excluded consisted of: (1) complete edentulism, (2) acute medical condition necessitating hospital admission in the past 3 months, (4) antibiotic treatment in the past 6 months, (5) periodontal therapy in the past 12 months, (6) severe cognitive impairment (Mini-Mental State Examination score <15) and (7) terminal illness with the expectation of death in less than 6 months.

## Data collection procedures:

## Sociodemographic, health information:

The trained research assistants carried out structured interviews on the age, gender, level of education, monthly income, smoking status (never/former/current), alcohol consumption, medical history (diabetes mellitus, hypertension, cardiovascular disease, arthritis), medication use and dental care utilization patterns. The body mass index (BMI) was determined using height and weight.

## Disability assessment:

Two validated instruments that were administered by trained geriatric nurses were used to assess functional disability. The Barthel Index was used to assess basic activities of daily living (ADL) in 10 domains, as feeding, bathing, grooming, dressing, bowel control, bladder control, toilet use, transfers, mobility and stairs (maximum score 0-100 with higher scores reflecting increased independence). The Lawton Instrumental Activities of Daily Living (IADL) scale comprised 8 items (telephone use, shopping, food preparation, housekeeping, laundry, transportation, medication management and financial management) (0-8 score, higher scores represented more independence).

Composite disability condition was classified as: (1) No disability: Barthel Index 100 and IADL 8; (2) Mild disability: Barthel Index 75-99 or IADL 6-7; (3) Moderate disability: Barthel Index 50-74 or IADL 4-5; (4) severe disability: Barthel Index <50 or IADL <4.

## Periodontal examination:

Two periodontists measured by two different periodontists (inter-examiner reliability κ=0.87) on dental clinics with standard dental chairs and lights via standard protocols. Examiners did not see the state of disability. All teeth except third molars were clinically measured on six sites (mesiobuccal, midbuccal, distobuccal, mesiolingual, midlingual and distolingual) using a UNC-15 periodontal probe to measure clinical parameters.

The parameters were recorded as follows (1) Probing depth (PD): distance between gingival margin and base of pocket; (2) Clinical attachment loss (CAL): distance between cementoenamel junction and gingival margin; (3) Bleeding on probing (BOP): present/absent within 30 seconds; (4) Gingival recession: distance between cementoenamel junction and gingival margin; (5) Plaque index: modified SilnessLoe index (0-3 scale); (6) Number of teeth present; (7) Number of teeth with mobility. Periodontitis was identified and categorized by the 2017 World Workshop on the Classification of Periodontal and Peri-Implant Diseases and Conditions: (1) No/mild periodontitis: CAL < 3 mm at all sites; (2) moderately periodontitis: interdental CAL 3 mm or more at 3 or more non-adjacent teeth or, buccal/oral CAL 3 mm or more at 3 or more teeth; (3) severe periodontitis: interdental CAL 5 mm or more at two or more non-adjacent teeth.

## Statistical analysis:

The analysis of data resorted to the use of STATA version 17.0 (StataCorp, Texas, USA). The tests of normality of continuous variables were performed with Shapiro-Wilk tests and Q-Q plots. Descriptive statistics were provided in terms of means with standard deviations in case of continuous variables and frequencies with percentage case in categorical variables. They used independent t-tests or the Mann- Whitney U tests when comparing two groups (continuous variables) and chi-square or Fisher exact tests when comparing two categories (categorical variables). The logistic regression analysis was used to test the relationship between disability and the outcome of periodontal diseases. Univariate analysis determined variables that were related to moderate-severe periodontitis (p<0.20 to qualify as variables). Multivariate logistic regression analyses were performed that controlled the possible confounding factors such as age, gender, education, income, smoking, diabetes, BMI and dental visit frequency. Calculations were done with odds ratios (OR) and 95% confidence intervals (CI). Linear regression evaluated disability scores-continuous periodontal parameters correlations. The level of statistical significance was established to be p<0.05 (two tailed).

## Results:

A total of 384 older adults (mean age 72.8 ± 6.4 years, range 65-89 years) completed all assessments. The sample comprised 198 females (51.6%) and 186 males (48.4%). Regarding disability status, 147 participants (38.3%) had no disability, 103 (26.8%) had mild disability, 89 (23.2%) had moderate disability and 45 (11.7%) had severe disability. Table 1 presents the sociodemographic and health characteristics stratified by disability status. Significant differences existed across disability categories for multiple variables. Table 2 presents periodontal disease prevalence and clinical parameters stratified by disability status. Significant progressive deterioration in periodontal health was observed with increasing disability severity. The prevalence of moderate-to-severe periodontitis increased from 52.4% in non-disabled individuals to 91.1% in those with severe disability (p<0.001). All clinical periodontal parameters demonstrated significant deterioration with increasing disability severity (p<0.001 for all comparisons). Table 3 presents results from logistic regression analysis examining the association between disability and moderate-to-severe periodontitis after adjusting for potential confounders. In the adjusted model, severe disability remained significantly associated with moderate-to-severe periodontitis (adjusted OR=3.84, 95% CI: 2.17-6.79, p<0.001), as did moderate disability (adjusted OR=3.24, 95% CI: 1.68-6.25, p<0.001) and mild disability (adjusted OR=1.87, 95% CI: 1.09-3.21, p=0.023). Other independent predictors included age, current smoking, diabetes mellitus and absence of recent dental visits. Linear regression analysis revealed significant negative correlations between Barthel Index scores and mean probing depth (β=-0.023, p<0.001), mean CAL (β=-0.028, p<0.001) and plaque index (β=-0.013, p<0.001), indicating that greater functional independence was associated with better periodontal health. Similar inverse relationships were observed for IADL scores.

## Discussion:

This research offers strong support to the fact that there is a strong independent role of functional disability and periodontal disease among community-dwelling older adults with strong dose-response relationship where the more the disability severity, the higher the prevalence and severity of periodontitis. The results have significant implications on geriatric models of care and preventive measures on oral health on the geriatric populations. The relationship between disability and periodontal disease that was observed is probable to show a combination of several interrelated mechanisms. The direct functional limitation is detrimental to economic oral hygiene such as effective toothbrushing and interdental cleaning ability [2]. People who have problems with fine motor control like the disabled, arthritics, or persons with mobility problems might find this hard because the fine motor control is needed to remove the plaque thoroughly as seen by the higher plaque index scores among the disabled in our study. Also, the functional disability is usually accompanied by cognitive impairment, which can undermine oral hygiene awareness and adherence [5]. In addition to mechanical obstacles of oral hygiene, disability is an impediment to professional dental services. Our results showed that only 26.7% of the severely disabled participants had dental check-ups in the last year as opposed to the 76.2% of the non-disabled population, which is in line with the previous studies that reported low dental care utilization amongst disabled populations [4]. This disparity is caused by transportation problems, physical access issues in dental centers, communication problems and financial problems. The lack of routine professional dental care gets rid of the possibility of early detection of periodontal disease, preventive measures and timely treatment. Also, the interaction between disability and periodontal disease can be a two-way and a cyclic one. Loss of teeth associated with periodontal disease impairs masticatory capability, which may be accompanied by changing the diet to soft and processed foods with low nutritional value [6]. Nutritional inadequacy especially low protein consumption is a contributor to sarcopenia and frailty which in turn worsen functional limitations [8]. Moreover, patient systemic effects of periodicontal disease chronic inflammatory burden can potentially contribute to muscle catabolism and function loss via inflammatory mediator products on skeletal muscle [3]. This reciprocal interaction explains the significance of combined interventions that target oral health and functional status.

Our adjusted models show the correlation with periodontal disease and diabetes, smoking and age as observed in the literature on risk factors of periodontal disease [11]. The increased chances of diabetes in the disabled sample (51.1% severely disabled and 23.1% non-disabled) indicates that there may be some confounding and mediation mechanisms that need to be examined in more detail by longitudinal design. Diabetes will lose wound healing and immune response and accelerate periodontal destruction [13] and periodontal inflammation can aggravate glycemic control, establishing an additional bidirectional relationship [8]. The clinical and population health importance of these results is tremendous. First, geriatric care involves implementing proactive oral health assessment and intervention by prompting disability assessment. Oral health screening during routine geriatric examination may help in the early detection of periodontal disease in the older population with functional impairment [14]. Second, oral hygiene assistance programmes in the case of those with functional restrictions, which may involve caregivers training, adaptive oral hygiene equipment, or home or residential delivery of oral hygiene services should be developed [15]. Third, the systemic barriers to dental care access among disabled older adults should be tackled by the healthcare policy with the help of mobile dental services, accessible facility design, transportation assistance programs and insurance coverage changes. The fact that the rate at which disabled participants visit dentists was significantly lower shows that there is a great demand to implement changes at the systemic level [16]. Fourth, the high-risk groups (both disabled and having other risk factors of periodontal disease, e.g. diabetes or smoking) should be the focus of prevention strategies that may be implemented in the form of multidisciplinary care coordination. There are some limitations of the study that should be mentioned. The cross-sectional nature of the design does not allow the causal inference of the directionality of the disability-periodontal disease relationship. Longitudinal studies are required to clarify the time relationships and determine whether the functional deterioration is prior to the periodontal deterioration or vice versa or whether they interact on each other or not. The convenience sampling can be a limitation in generalizability where, however, recruitment across varied community environments diversified the samples. The confounding factors caused by unknown factors (medication effects, dietary, susceptibility of a person to the disease, etc.) may not be eliminated [17]. It did not discriminate between the various forms of disabilities (physical, cognitive, sensory) and this could have had varied relations with periodontal disease in different ways. The proposed study should be followed up by investigations into the type-specific relationships between disability and the search of specific intervention strategies. Also, oral health behaviors that were not assessed fully (e.g. the frequency of visits to the dentist, frequency of brushing, frequency of flossing, frequency of using mouthwash) could be partial mediators of the disability-periodontal disease correlation. Lastly, although we used standardized periodontal examination procedures, the variability of examiners cannot be fully removed even when they are calibrated [18].

## Conclusion:

Functional disability has a strong independent and dose-related relationship with the prevalence and severity of periodontal disease in community-based elderly adults and a severely disabled person shows almost four times the likelihood of moderate-severe periodontitis after correction of confounding factors. These results underline the urgent need to pursue integrated geriatric-dental care models, proactive oral health measures to address the needs of functionally impaired older adults and systematic endeavors to address dental care access disparity in disabled people to avert synergistic decline in the function and oral health conditions.

## Figures and Tables

**Table 1:** Sociodemographic and health characteristics by disability status (n=384)

**Characteristic**	**No Disability (n=147)**	**Mild Disability (n=103)**	**Moderate Disability (n=89)**	**Severe Disability (n=45)**	p-value*
Age (years)	70.2 ± 4.8	72.6 ± 5.9	74.8 ± 6.7	78.4 ± 7.2	<0.001
Female gender, n (%)	68 (46.3%)	54 (52.4%)	48 (53.9%)	28 (62.2%)	0.189
Education ≤8 years, n (%)	42 (28.6%)	47 (45.6%)	56 (62.9%)	34 (75.6%)	<0.001
Low income**, n (%)	35 (23.8%)	41 (39.8%)	51 (57.3%)	31 (68.9%)	<0.001
Current smoker, n (%)	18 (12.2%)	24 (23.3%)	28 (31.5%)	17 (37.8%)	<0.001
Diabetes mellitus, n (%)	34 (23.1%)	32 (31.1%)	38 (42.7%)	23 (51.1%)	<0.001
Hypertension, n (%)	76 (51.7%)	64 (62.1%)	61 (68.5%)	34 (75.6%)	0.006
Arthritis, n (%)	41 (27.9%)	48 (46.6%)	57 (64.0%)	32 (71.1%)	<0.001
BMI (kg/m^2^)	24.8 ± 3.6	24.2 ± 3.9	23.1 ± 4.2	21.8 ± 4.7	<0.001
Dental visit in past year, n (%)	112 (76.2%)	63 (61.2%)	39 (43.8%)	12 (26.7%)	<0.001
Barthel Index score	100.0 ± 0.0	88.5 ± 7.3	64.2 ± 9.8	38.7 ± 8.4	<0.001
IADL score	8.0 ± 0.0	6.4 ± 0.8	4.6 ± 0.7	2.8 ± 1.2	<0.001
*One-way ANOVA or chi-square test;
*Monthly income <$1000;
Values expressed as mean ± SD or n (%)

**Table 2:** Periodontal disease prevalence and clinical parameters by disability status

**Periodontal Parameter**	**No Disability (n=147)**	**Mild Disability (n=103)**	**Moderate Disability (n=89)**	**Severe Disability (n=45)**	p-value*
Periodontitis severity, n (%)					<0.001
No/mild periodontitis	70 (47.6%)	31 (30.1%)	16 (18.0%)	4 (8.9%)	
Moderate periodontitis	58 (39.5%)	51 (49.5%)	43 (48.3%)	19 (42.2%)	
Severe periodontitis	19 (12.9%)	21 (20.4%)	30 (33.7%)	22 (48.9%)	
Moderate-to-severe periodontitis	77 (52.4%)	72 (69.9%)	73 (82.0%)	41 (91.1%)	<0.001
Mean probing depth (mm)	2.4 ± 0.6	2.9 ± 0.7	3.3 ± 0.8	3.9 ± 0.9	<0.001
Sites with PD ≥4 mm (%)	12.3 ± 8.4	18.7 ± 11.2	26.4 ± 14.6	35.8 ± 17.3	<0.001
Mean CAL (mm)	2.8 ± 0.9	3.6 ± 1.1	4.2 ± 1.3	5.1 ± 1.6	<0.001
Sites with CAL ≥5 mm (%)	8.7 ± 7.2	15.4 ± 10.8	23.6 ± 13.4	32.7 ± 16.9	<0.001
Bleeding on probing (%)	28.4 ± 15.6	36.2 ± 18.3	43.8 ± 19.7	51.6 ± 21.4	<0.001
Plaque index	1.2 ± 0.5	1.6 ± 0.6	2.0 ± 0.7	2.4 ± 0.6	<0.001
Number of teeth present	22.4 ± 4.8	20.1 ± 5.3	17.6 ± 5.9	14.8 ± 6.2	<0.001
Teeth with mobility (%)	4.2 ± 5.3	7.8 ± 7.6	12.4 ± 9.8	18.3 ± 11.4	<0.001
One-way ANOVA or chi-square test;
PD = probing depth;
CAL = clinical attachment loss;
Values expressed as mean ± SD or n (%)

**Table 3:** Logistic regression analysis of factors associated with moderate-to-severe periodontitis

**Variable**	**Unadjusted OR (95% CI)**	**p-value**	Adjusted OR* (95% CI)	**p-value**
**Disability status**				
No disability	1.00 (reference)	-	1.00 (reference)	-
Mild disability	2.13 (1.28-3.54)	0.004	1.87 (1.09-3.21)	0.023
Moderate disability	4.18 (2.26-7.73)	<0.001	3.24 (1.68-6.25)	<0.001
Severe disability	9.14 (3.12-26.78)	<0.001	3.84 (2.17-6.79)	<0.001
Age (per year increase)	1.08 (1.04-1.12)	<0.001	1.06 (1.02-1.10)	0.002
Male gender	1.76 (1.14-2.72)	0.011	1.58 (0.98-2.54)	0.061
Education ≤8 years	2.34 (1.48-3.69)	<0.001	1.42 (0.84-2.40)	0.187
Low income	2.87 (1.73-4.76)	<0.001	1.68 (0.95-2.98)	0.076
Current smoking	3.42 (1.84-6.36)	<0.001	2.94 (1.52-5.68)	0.001
Diabetes mellitus	2.56 (1.58-4.15)	<0.001	2.18 (1.29-3.68)	0.004
BMI (per unit increase)	0.92 (0.87-0.98)	0.007	0.94 (0.88-1.00)	0.053
No dental visit in past year	3.28 (2.04-5.27)	<0.001	2.41 (1.44-4.03)	0.001
Adjusted for all variables listed in the table;
OR = odds ratio;
CI = confidence interval
